# Measuring Health Spillovers for Economic Evaluation: A Case Study in Meningitis

**DOI:** 10.1002/hec.3259

**Published:** 2015-10-14

**Authors:** Hareth Al‐Janabi, Job Van Exel, Werner Brouwer, Caroline Trotter, Linda Glennie, Laurie Hannigan, Joanna Coast

**Affiliations:** ^1^ Health Economics Unit, School of Health and Population SciencesUniversity of BirminghamBirminghamUK; ^2^ Institute of Health Policy and ManagementErasmus University RotterdamRotterdamNetherlands; ^3^ Disease Dynamics Unit, Department of Veterinary MedicineUniversity of CambridgeCambridgeUK; ^4^ Meningitis Research FoundationBristolUK

**Keywords:** economic evaluation, health valuation, informal care, EQ‐5D, spillovers

## Abstract

The health of carers and others close to the patient will often be relevant to economic evaluation, but it is very rarely considered in practice. This may reflect a lack of understanding of how the spillover effect of illness can be appropriately quantified. In this study we used three different approaches to quantify health spillovers resulting from meningitis. We conducted a survey of 1218 family networks affected by meningitis and used regression modelling to estimate spillover effects. The findings show that meningitis had long‐term effects on family members' health, particularly affecting the likelihood of family members reporting anxiety and depression. These effects extended beyond a single close family member. These findings suggest that vaccinating against meningitis will bring significant health benefits not just to those that might have contracted the illness but also to their family networks. In methodological terms, different approaches for quantifying health spillovers provided broadly consistent results. The choice of method will be influenced by the ease of collecting primary data from family members in intervention contexts. © 2015 The Authors. *Health Economics* published by John Wiley & Sons Ltd.

## 1 Introduction

When an individual develops a serious illness, it is likely to be a time of great stress for those close to them. Family members may worry and grieve, feel guilt and uncertainty, and undertake increased caring responsibilities. Consequently, healthcare interventions may improve not only patients' lives but also the lives of those close to the patient. These broader benefits of healthcare may include health benefits for the patient's family. Health benefits may stem from family members' reduced anxiety or from freeing them from physically draining caring responsibilities (Christakis and Iwashyna, 2003, Pinquart and Sorensen, 2003, Bobinac *et al.*, 2011). Despite many calls for these wider health benefits (which we term ‘health spillovers’ in this paper) to be considered in economic evaluations (Brouwer *et al.*, 1999, Al‐Janabi *et al.*, 2011, National Institute for Health and Care Excellence (NICE), 2013, Gold *et al.*, 1996), they are almost always ignored (Goodrich *et al.*, 2012). In part, this is likely to reflect a lack of awareness of how health spillovers can be quantified for economic evaluation.

Formal consideration of health spillovers in economic evaluation has been advocated for some time. The 1996 US Panel on Cost‐Effectiveness suggested that “*In a CEA…all health effects that flow from it [the intervention] are counted. Health effects include both benefits and harms, even when these occur in people who are not the intended recipients of the intervention.*” (Gold *et al.*, 1996). In their latest guidance, the UK's National Institute for Health and Care Excellence also highlights the relevance of health spillovers, commenting that economic evaluations should consider: “*…all direct health effects, whether for patients or, when relevant, carers*” (NICE, 2013). Consideration of health spillovers is needed to understand the total health benefits of healthcare interventions. Furthermore, health spillovers matter in practice, as inference about the most cost‐effective treatment strategy can rest on whether health spillovers are considered (Bilcke *et al.*, 2009, Krol *et al.*, 2015).

In health economics, much of the attention on spillover effects has focused on the wider welfare (as opposed to ‘health’) impacts of healthcare consumption. Early work by Culyer (Culyer, 1989, Culyer and Simpson, 1980, Culyer, 1971) showed how healthcare consumption could generate welfare gains (‘caring externalities’) for others in society. Subsequent empirical work has shown that caring externalities extend widely in scope (Jacobsson *et al.*, 2005) and can be large in magnitude (Drummond *et al.*, 1991, Basu *et al.*, 2010, Hurley and Mentzakis, 2013, Prosser *et al.*, 2004). Some authors have proposed conceptual frameworks to take these wider welfare effects into account in economic evaluation (Basu and Meltzer, 2005).

Yet it is a narrower interpretation of health spillover that is relevant to most applied economic evaluations. In practice, the focus is usually on maximising some measure of health output (often quality‐adjusted life years (QALYs)) rather than welfare *per se* (Brouwer *et al.*, 2008, Coast *et al.*, 2008, Drummond *et al.*, 2005). Although health maximisation rules‐out wider welfare effects, it rules‐in wider health effects. A review of perspectives taken in economic evaluation highlights that health effects on carers are relevant to consider, when maximising health, whether one takes a societal or a healthcare perspective to the economic evaluation (Claxton *et al.*, 2010).

There is a growing literature on the wider health effects of illness. This research demonstrates, for example, that many health conditions affect the health status of close family members. For example, studies have shown that patient disabilities affect family members' health status across a range of childhood conditions (Tilford *et al.*, 2005, Poley *et al.*, 2012, Yamazaki *et al.*, 2005, Wittenberg *et al.*, 2013) and adult conditions (Davidson *et al.*, 2008, Peters *et al.*, 2011, Gallagher and Mechanic, 1996, Coe and Van Houtven, 2009). There is also a wide literature that shows that informal carers' health suffers, as the degree of disability and needs of the patient increases (Brouwer *et al.*, 2004, Argimon *et al.*, 2004, Bobinac *et al.*, 2011). These studies strongly suggest that healthcare interventions that treat a patient's illness would have health spillover benefits to their close family members. However, there are two gaps which need addressing to incorporate such evidence in economic evaluation. First, it is not obvious how the health spillover benefits of interventions can be quantified for use in economic evaluation. Second, the focus of existing research is mainly on the patient's family caregiver, and it is not clear whether there is a need to consider health benefits beyond a single close family member.

The research reported here aimed to address these gaps. We used a case study of meningitis vaccination to illustrate how health spillovers could be quantified for use in economic evaluation. Our specific objectives were to (i) identify whether health spillovers were likely to result from vaccination against meningitis; (ii) establish whether the spillover effect extends to multiple family members of the same patient; and (iii) compare methods for quantifying health spillovers for consideration in economic evaluation.

## 2 Meningitis Case Study

**Figure 1:**
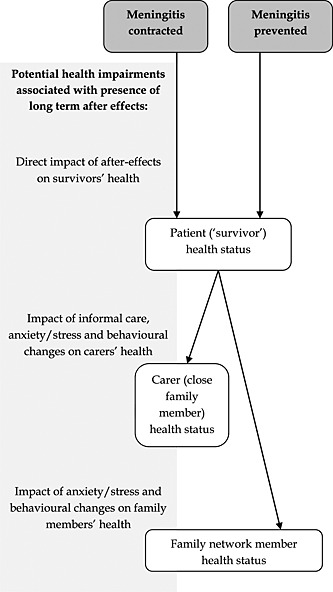
Framework for examining health spillovers arising from the prevention of meningitis

Meningitis is an acute, potentially life‐threatening infection of the membranes surrounding the brain and spine. It is most common in very young children, although it can be contracted at any age. Mortality during the acute phase varies by cause, age, and country with around 2–11% of people affected dying in the UK (Visintin *et al.*, 2010). Survivors often make a full recovery from the infection, but evidence suggests that around 20% of individuals who contract bacterial meningitis develop one or more after‐effects (Viner *et al.*, 2012, Edmond *et al.*, 2010). These after‐effects include cognitive problems, seizures, hearing loss, motor limitations, amputations, vision problems, and behavioural problems. The after‐effects are not only likely to impact on the lives of the survivors but also, through the potential stress, additional care requirements, and changes in behaviours (such as self‐care and healthcare use) on the lives of their close family members. We have outlined a simple diagram showing the potential scope of health effects arising from the disabling after‐effects of meningitis (Figure 1).

Many causes of meningitis can be largely prevented by vaccination. Vaccination saves lives, but a major benefit is also that it prevents individuals contracting meningitis and living a lifetime with the disabling after‐effects of the illness. Thus vaccination would shift many potential patients and their families from the first pathway in Figure 1 to the second pathway (prevention). Some causes of meningitis, notably ‘MenB’, have proved difficult to vaccinate against, although promising vaccines have recently been developed. From a policy perspective, it is important to know whether new vaccines are cost‐effective, and this requires a calculation of the likely health benefits. If health benefits are likely to accrue, not just to potential patients but also to their family members, then this may be relevant to decision‐makers.

## 3 Methods

### 3.1 General approach

To examine the presence of health spillovers arising from the long‐term effects of meningitis, we conducted a prospective survey of families affected by meningitis. This survey provided data on the health outcomes of survivors and their close family members. We then used three methods to quantify the scale of health spillovers arising from the long‐term after‐effects of meningitis.

Method 1 uses a comparison group to identify the health decrement associated with after‐effects. The spillover effect is the difference in health status between family members in the group ‘exposed’ to after‐effects and family members in the comparison group. In our study the comparison group was family members who were not exposed to the long‐term after‐effects of meningitis.

Method 2 uses regression analysis to express a family member's health status as a function of the survivor's health status. The spillover effect is given by the coefficient on variable(s) representing the survivor's health status. This provides an estimate of what we term the ‘relative’ spillover effect, as it shows the change in an average family member's health status *relative* to a change in the survivor's health status.

Method 3 is an extension of the regression‐based approach employed in Method 2, to explicitly examine the spillovers in a survivor's wider family network. To examine these wider spillovers we collected data from multiple members of the same family network. Separate regression analyses were conducted in different groups of family members, and the findings used to provide estimates of what we term the ‘aggregate’ spillover effect.

### 3.2 Data collection: ‘family impact of meningitis study’

We identified family members of people affected by meningitis from the membership records of a large meningitis 1Septicaemia was included as well as both conditions can co‐exist and be prevented through vaccination. For brevity, and in view of the small number of cases that were solely septicaemia, we use ‘meningitis’ to refer to both. charity (Meningitis Research Foundation (MRF)) in the UK. Family members were included if they were 13 years old or over, and were excluded if they had contracted meningitis themselves, were not close to someone who had contracted meningitis, or if the person affected by meningitis had died.

We developed a survey questionnaire for family members to complete 2In this study we describe all respondents as ‘family members’, although a very small proportion were in fact close friends. and refined the questionnaire content using a qualitative focus group (*n* = 6) and small postal pilot (*n* = 30) with MRF members. In the questionnaire, the health status of family members and survivors was measured using the EQ‐5D‐5L (Herdman *et al.*, 2011), the generic health status tool advocated by National Institute for Health and Care Excellence (NICE) for calculating QALYs (NICE, 2013). Family members reported the health status and the presence of any long‐term after‐effects from meningitis for the survivors. Proxy reports were necessary because of the large number of survivors that would be unable to self‐report their health because of their age or disability. In addition, we included a number of questions about the meningitis infection and socio‐demographic circumstances.

Our sample size was based on previous studies of the association between patient and carer health status (Bobinac *et al.*, 2011, Tilford *et al.*, 2005). These studies indicated that a relative spillover effect of 0.1 in family member health status in a regression model was plausible. To detect such an effect with 80% probability at *p* = 0.05 required at least 787 respondents. Assuming a response rate to the survey of 25%, we needed an initial sample frame of 3148 members. From the MRF database, we identified 3417 potentially eligible members. Rather than exclude 269 members, all members were included. Questionnaires were included in a survey pack with information about the study and a pre‐paid reply envelope and posted to all eligible members of the MRF in May 2012.

To investigate whether spillovers were present in multiple members of the same family network, we sent two questionnaires to each member. The named recipient was asked to complete the first questionnaire and pass the second questionnaire on to a second person close to the survivor. A reminder postcard was sent to all members after one week, and a reminder letter was sent to all non‐responders after four weeks. We entered data into a secure database, with 5% of questionnaires being double entered to verify the accuracy of data entry. The study protocol was approved by the University of Birmingham's Life and Health Sciences Ethical Review Committee (ERN_11‐0191).

### 3.3 Estimating the absolute health spillover effect on family members (Method 1)

We split family members into those exposed to after‐effects and those who were unexposed to examine whether family members' health status was influenced by the after‐effects of meningitis. We used survey responses to determine whether family members were exposed to one or more of 20 possible after‐effects. Pre‐specified after‐effects were determined by literature (Edmond *et al.*, 2010) discussion with experts, and the aforementioned focus group with MRF members. Additionally a number of family members reported the presence of after‐effects that were not covered by the list. These additional after‐effects were reviewed for plausibility by three of us (two of whom have expertise in the epidemiology of meningitis) and individually coded for inclusion or exclusion. Family members were included in the exposed group if they recorded the survivor as having one or more long‐term after‐effects from meningitis.

To estimate family members' health status on a 0 (death) to 1 (full health) scale, responses to the EQ‐5D‐5L questionnaire were scored using interim value sets for the UK (Van Hout *et al.*, 2012). *T*‐statistics were used to identify whether the health status (EQ‐5D‐5L) of family members exposed to after‐effects differed from those who were not exposed. We used univariable logistic regressions to identify whether family members were more likely to report any problems (as opposed to no problems) in the individual domains of the EQ‐5D‐5L in the presence of after‐effects in the survivors. To set these findings in context, we repeated the analysis to examine how survivors themselves were affected by the after‐effects of meningitis.

### 3.4 Estimating the relative health spillover effect on family members (Method 2)

To estimate the relative spillover effect we modelled the health status of family members as a function of the health status of survivors, controlling for other contextual factors. We adapted the approach used by Bobinac *et al.* to estimate spillover effects on informal carers (Bobinac *et al.*, 2010, Bobinac *et al.*, 2011), by expressing the health status of family members (*H_f_*) as *H_f_* = *f*(*H_p_*, *X_f_*, *X_p_*, *C*) where *H_p_* is the health status of patients (survivors), *X_f_* is a vector of characteristics of the family member, *X_p_* is a vector of characteristics of the survivor, and *C* is a vector of contextual characteristics. When this model is estimated through regression analysis, the coefficient on the *H_p_* variable provides the relative health spillover effect. 3It is important to note that we are focusing on spillover effects as associations between the health changes in survivors and their family members. We do not seek to demonstrate, nor require, that causality runs solely from patient health status to family member health status. In the rest of the paper, we refer to this coefficient as the ‘spillover coefficient’.

In the regression models, the health status of the family members (*H_f_*, dependent variable) and survivors (*H_p_*, independent variable) was measured using EQ‐5D‐5L index scores. We attempted to control for other factors that could affect family member and survivor health status and thus confound any true spillover effect (Manski, 1993). In terms of socio‐demographic characteristics, we controlled for the family member's age, sex, education level and employment status, and survivor's age and sex. We additionally controlled for a number of contextual factors that may influence both family member and survivor health status. These were (i) the time elapsed since the initial infection, as both survivor and family member may adapt to the effects of the meningitis; (ii) the presence of a biological relationship, as both may have a genetic predisposition to poor/good health; (iii) co‐residence, as there may be contemporaneous environmental exposures; and (iv) the number of people in the family member's household, as the health of both groups may be affected by common social support networks. The regression models were estimated in the sub‐sample of family members exposed to after‐effects of meningitis. Analyses were conducted including individuals with complete data across all variables considered for the regression modelling.

We checked that a significant spillover effect was not simply an artefact of the fact that family members reported the survivor's health status as well as their own. To do this we used data from family networks with two family members responding. We replaced the first family member's proxy report of survivor health status with the second family member's proxy report (and vice versa). We compared the significance and magnitude of the spillover coefficient when independent ratings of survivor health status were used, with the spillover coefficient when non‐independent ratings of survivor health status were used.

In common with other studies of health spillovers (Coe and Van Houtven, 2009, Bobinac *et al.*, 2011, Bobinac *et al.*, 2010, Tilford *et al.*, 2005) all regression models were estimated using ordinary least squares regression. We initially ran a univariable regression (of *H_f_* on *H_p_*) and then introduced family member characteristics, survivor characteristics, and contextual characteristics sequentially into the regression model to examine what, if any, impact this had on the significance and magnitude of the spillover coefficient. Data analysis was conducted using Stata v12. Standard errors in the regression models were adjusted for clustering of the observations at the family level. Non‐linear relationships between the dependent variable and the four continuous independent variables in the model (family member health status, survivor age, family member age, and time elapsed since infection) were explored using graphical and statistical methods. The Ramsey Regression Equation Specification Error Test (RESET) test was used to identify mis‐specification of the functional form of the variables.

### 3.5 Estimating the aggregate health spillover effect (Method 3)

To examine whether spillovers were present only in closest family members, we examined the presence of spillovers in family networks where two family members responded. We first re‐estimated the regression model in the sub‐sample of family members deemed closest 4As there is not an established way of assessing the closest person in a family network, we used the data collected on co‐residence, social contact, relationship, and employment status to try to classify which family member could be deemed ‘closer’ to the survivor. We defined the closer family member as the one who was co‐resident or reported more social contact with the survivor. If family members tied on these criteria we classified the family member as closer if they were a closer relation or reported a lower level of employment (potentially indicating more time at home with the survivor). Some parents tied on all criteria. In these cases we classified mothers as closer than fathers (Mallers et al., 2010)). and then in the sample of family members deemed second closest. We took a significant spillover coefficient in the second regression model to indicate spillover effects that extended beyond the closest family network member. We used the same specifications for the regression models as previously.

We also re‐ran the regression models within specific groups of family members (parents, partners, grandparents, children, and siblings) as an alternative way of examining the potential for multiple spillovers within a family network. The sign, significance, and magnitude of the spillover coefficients were used to check whether the health status of different groups of relatives were associated with the health status of the survivors.

## 4 Results

### 4.1 Survey response

**Figure 2:**
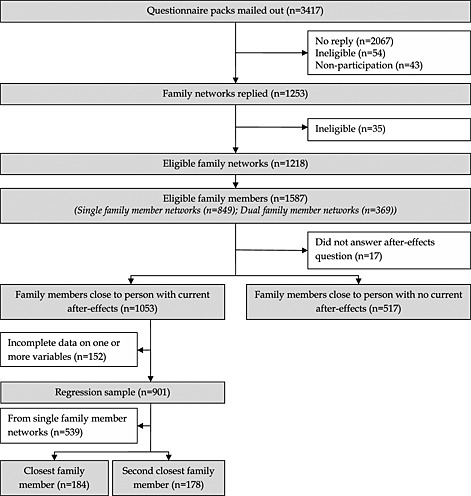
Response to the survey and inclusion at different stages of the analysis

Figure 2 displays the response to the survey and the number of family members included at each stage of the analysis. Thirty‐seven per cent of households contacted responded to the survey, 97% of whom were eligible to be included. The most common reason for ineligibility was that the person affected by meningitis had died, and the most common reason cited for non‐participation was that the meningitis episode was too long ago. Thirty‐one per cent of the responding households provided responses from two family members. In total, there were responses from 1587 eligible family members of 1218 survivors.

The sample of the respondents (‘whole sample’; Table 1) comprised a majority of female respondents. Most respondents were parents of the survivors (76%, *n* = 1192) although a number of partners (6%, *n* = 102) and grandparents (9%, *n* = 137) responded. The initial meningitis infection occurred an average of 12 years ago, and survivors were now aged 23 years old on average.

**Table 1:** Descriptive statistics of the participants in the study

Characteristic	Whole sample (*n* = 1587)	Regression sub‐sample (*n* = 901)
Family member		
Sex (female, %)	74%	77%
Age (years, mean (SD))	51 (13)	50 (12)
Health status (EQ‐5D‐5L, mean (SD))	0.88 (0.16)	0.88 (0.16)
Education (degree, %)	43%	41%
Employment (full‐time, %)	35%	35%
Survivor		
Sex (female, %)	46%	46%
Age (years, mean (SD))	23 (16)	24 (17)
Health status (EQ‐5D‐5L, mean (SD))	0.84 (0.26)	0.77 (0.27)
After‐effects reported (%)	67%	100%
Time since infection (years, mean (SD))	12 (7)	12 (8)
Context		
Biological relation (%)	92%	91%
Co‐resident (yes, %)	61%	65%
Adults in house (mean)	2.27	2.26
Children in house (mean)	0.98	0.98

### 4.2 Impact of after‐effects on family members' health status

Sixty‐seven per cent (*n* = 1053) of family members reported at least one after‐effect from meningitis in the survivor. The most commonly reported after‐effects were behavioural or emotional problems (28%), mild/moderate learning disabilities (16%), scarring or tissue damage (14%), balance problems (13%), and speech or language problems (11%).

The presence of after‐effects in survivors was associated with lower health status for family members. The mean difference in EQ‐5D‐5L scores between exposed family network members and controls was 0.041 (95% CI: 0.024 to 0.058) (Table 2). Further analysis (available on request) shows that this decrement is fairly constant with respect to time since the initial infection. This suggests that for the purposes of economic evaluations, it may be reasonable to interpret the 0.041 figure as an annual QALY decrement for close family members.

**Table 2:** A comparison of the health status of the family members and survivors exposed to after‐effects of meningitis compared with those who were unexposed

Characteristic	Exposed (*n* = 1053)	Unexposed (*n* = 517)
Family member		
Health status (EQ‐5D‐5L, mean)	0.87***	0.91
Mobility problems (%)	14%	13%
Self‐care problems (%)	3%	2%
Usual activities problems (%)	14%*	10%
Pain/discomfort problems (%)	33%*	27%
Anxiety/depression (%)	40%***	23%
Survivor		
Health status (EQ‐5D‐5L, mean)	0.78***	0.97
Mobility problems (%)	24%***	1%
Self‐care problems (%)	19%***	1%
Usual activities problems (%)	37%***	3%
Pain/discomfort problems (%)	38%***	4%
Anxiety/depression (%)	46%***	9%

*p < 0.05, **p < 0.01, ***p < 0.001.

Family members exposed to the after‐effects of meningitis had 2.3 (95% CI: 1.8 to 2.9) times higher odds of reporting anxiety or depression, 1.4 (95% CI: 1.0 to 2.0) times higher odds of problems with usual activities, and 1.3 (95% CI: 1.0 to 1.6) times higher odds of pain or discomfort compared with family members of survivors without after‐effects. The health status of survivors with after‐effects from meningitis was significantly worse than those without after‐effects with a mean difference in EQ‐5D‐5L between the two groups of 0.19 (95% CI: 0.17–0.22)).

### 4.3 The relative health spillover effect on family members

Regression models to estimate the spillover coefficient were estimated using 901 out of the 1053 family members close to someone with after‐effects (Table 3). The model estimations indicate a positive spillover coefficient of 0.16 (*p* < 0.001). The direction, statistical significance and magnitude of the spillover coefficient was unaffected by the introduction of variables to control for characteristics of the family member, survivor, or illness context (model 1 and model 2). Model 2 indicates that in addition to being positively associated with survivor health status, family member health status was positively associated with the family member being male, young, employed, biologically related to the survivor, and close to survivor who was male, older, and had a more recent infection. Investigations of the presence of non‐linear relationships between the independent variables and the dependent variable provided no evidence for including non‐linear terms in the regression model. Ramsey RESET tests showed no mis‐specification.

**Table 3:** Regression model estimates of the relative spillover effect on family members' health status (*n* = 901)

Variables	Model 1 (univariable)	Model 2 (multivariable)
Survivor		
Health status (EQ‐5D‐5L)	0.16***	0.16***
Sex (male)	‐	0.025*
Age (years)	‐	0.0011**
Time since infection (years)	‐	−0.0018*
Family member		
Sex (male)	‐	0.032*
Age (years)	‐	−0.0019***
Education		
16	‐	0.023
18	‐	0.033
Degree	‐	0.041
Employment (full‐time)	‐	0.031**
Context		
Non‐biological relation	‐	−0.049*
Co‐resident	‐	0.0085
Adults sharing house	‐	0.0096
Children sharing house	‐	0.010
	*R* ^2^ = 0.072	*R* ^2^ = 0.163

*p < 0.05, **p < 0.01, ***p < 0.001.

The effect of re‐estimating the regression model using independent ratings of the survivors' health status is shown in Table 4. The positive and statistically significant spillover coefficient remained when independent ratings of survivor health status were used (model 3). This suggested that using the same individuals to rate their own health status and the survivor's health status did not appear to bias the results (i.e. model 3 and model 4 provide identical estimates of the spillover coefficient).

**Table 4:** Robustness test of regression modelling using independent proxy ratings of survivors' health status

Variables	Model 3 (independent proxy)	Model 4 (base case)
Survivor		
Health status (EQ‐5D‐5L)	0.16***	0.17***
Sex (male)	0.0055	0.0061
Age (years)	0.00072	0.00071
Time since infection (years)	−0.0025*	−0.0025*
Family member		
Sex (male)	0.016	0.0016
Age (years)	−0.0014	−0.0014
Education		
16	−0.016	−0.0095
18	0.0012	0.0016
Degree	0.0011	0.0014
Employment (full‐time)	0.038*	0.036*
Context		
Non‐biological relation	−0.044	−0.041
Co‐resident	0.0019	0.0019
Adults sharing house	−0.0093	−0.0083
Children sharing house	0.019*	0.019*
*R* ^2^	0.206	0.211
Observations	326	326

Note: Individuals are included in this analysis only if they are from dual family networks where both family members provided full data across all relevant variables.

*p < 0.05, **p < 0.01, ***p < 0.001.

### 4.4 Aggregate health spillovers

Three hundred sixty‐two respondents from family networks with two respondents provided full data across all variables considered in the regression model. These respondents were allocated to be in either the sub‐sample of closest (*n* = 184) or second closest (*n* = 178) family members. The spillover coefficient in the first sample (closest family members) was 0.18 (95% CI: 0.10 to 0.27), and in the second sample (second closest family member) it was 0.11 (95% CI: 0.04 to 0.18) (Table 5). Model 6 indicates the presence of a statistically significant effect on the second closest family members and therefore that health spillovers may extend to more than one close family member. The lower spillover coefficient in model 6 (0.11) relative to model 5 (0.18) suggests that the spillover effect declines with increasing social distance from the survivor.

**Table 5:** Estimation of the relative spillover effect in closest and second closest family members

Variables	Model 5 (closest family member)	Model 6 (second closest family member)
Survivor		
Health status (EQ‐5D‐5L)	0.18***	0.11**
Sex (male)	0.025	0.016
Age (years)	0.00093	0.0013
Time since infection (years)	−0.0025	−0.0036*
Family member		
Sex (male)	0.0050	0.031
Age (years)	−0.0022	−0.00058
Education		
16	−0.0092	−0.047
18	0.017	‐0.029
Degree	0.025	‐0.032
Employment (full‐time)	0.024	0.041
Context		
Non‐biological relation	−0.048	−0.012
Co‐resident	‐0.031	0.032
Adults sharing house	‐0.032	0.012
Children sharing house	0.0039	0.026*
*R* ^2^	0.168	0.173
Observations	184	178

*p < 0.05, **p < 0.01, ***p < 0.001.

Regression models run within specific groups of family members suggested that different family relations may be affected by the after‐effects of meningitis. The spillover coefficient was positive and significant (*p* < 0.05) for parents (0.14), siblings (0.35), and partners (0.24) (Table 6). In this sample, spillover coefficients within sub‐samples of grandparents and children of the survivors were non‐significant at the 5% level.

**Table 6:** Regression estimates of the spillover coefficients by relation to survivor

Regression sample	Spillover coefficient
Model 7: parents (*n* = 692)	0.14***
Model 8: siblings (*n* = 31)	0.36*
Model 9: partners (*n* = 72)	0.24***
Model 10: grandparents (*n* = 59)	0.14
Model 11: children (*n* = 31)	−0.05

Other relations are omitted because of the small number (<10) of observations.

All regression models are adjusted for the potential confounding variables outlined in Model 2 (Table 3).

*p < 0.05, **p < 0.01, ***p < 0.001.

## 5 Discussion

This study illustrates how health spillovers can be measured for use in economic evaluation. Using a case study in meningitis, we found that long‐term morbidity from meningitis resulted in health losses to survivors' family members (in addition to the survivors themselves). This implies that there will be spillover benefits from vaccinating against meningitis for patients' family networks. These health spillovers were quantified in different ways. The absolute benefit of avoiding long‐term morbidity in a survivor was estimated to be 0.041 QALYs per close family member, per year. Regression modelling showed that, on average, family member health status would change by 16% of the change in survivor health status. We found that spillover effects were also likely to extend beyond the closest family members. Later in this section we discuss how these different spillover estimates could be incorporated within an economic evaluation.

The magnitude of the spillovers estimated in our study is consistent with findings from some other studies (Bobinac *et al.*, 2011, Tilford *et al.*, 2005). However, spillovers are unlikely to be consistent across contexts. A recent review suggests substantial variation in the likelihood of spillover by context (Wittenberg and Prosser, 2013). Recent evidence indicates that spillover impacts are likely to be greatest when dealing with childhood and mental health conditions (Wittenberg *et al.*, 2013, Lavelle *et al.*, 2014). As many of the survivors in this study were children with psychological problems, it seems likely that our spillover estimates would be at the higher end of the spectrum. In terms of the magnitude of the spillover, a 0.041 difference on the EQ‐5D‐5L is in line with reported minimally important differences in preference‐based measures of health status (Luo *et al.*, 2010). Furthermore, as noted in the results, the spillover effect is likely to persist for many years after the initial infection. The total health spillover, in terms of QALYs per family member, is therefore likely to be much greater.

Our finding that spillovers extend beyond the closest family member and decline with increased social distance is consistent with research on social networks. This literature suggests that many health‐related phenomena spread in social networks (Christakis and Fowler, 2013). Studies show that the ‘force’ of the spread declines with social distance from the central individual in the study (Christakis and Fowler, 2013). We found that the after‐effects of meningitis appear to have ‘contagious’ negative effects on family members' health status. As vaccination would remove the source of the contagion, vaccination would generate health benefits that extend beyond the immediate beneficiaries. At first glance it might seem surprising that health benefits to close family members would potentially exceed 29% 5i.e. 18% in the closest family member (based on the 0.18 spillover coefficient) and then 11% in the second closet family member (based on the 11% spillover coefficient). of the direct benefit to patients themselves, given the debilitating impact of after‐effects on the lives of the survivors. But, it is important to note that the scale of health spillovers is a product of both the impact on family members and the number of family members affected. Thus, when effects on individual family members are aggregated over the wider family network they may become quite large. Furthermore, this study focuses on spillovers in terms of family members' health status. If spillovers also occur in terms of, for example, families' labour force participation, child‐rearing decisions, and social activities, the total effect on families will be greater than estimated here.

We used three approaches to quantify health spillovers. The ‘absolute spillover’ approach (Sections 3.3 and 4.2) is likely to work best when outcome data can be collected as part of a controlled trial of the intervention of interest. Trials would allow direct evidence of the effect of the intervention on family members to be collected alongside evidence of the effect on patients. The ‘relative spillover’ approach (see Sections 3.4 and 4.3), on the other hand, is more suited to contexts where data on the effect of the intervention on family members cannot be collected, for example, because a trial is not feasible for resource, ethical or practical reasons. In this case, observational data may be used to model the degree to which changes in patient health may spillover to affect the health status of close family members. Finally, an estimation of the aggregate spillover (see Sections 3.5 and 4.4) to the whole family network may be the most desirable, in theory. However, it will be challenging to aggregate spillover effects across the family with precision. In practice, characterising the total health benefits from interventions is likely to require a mixture of primary research and modelling work. In the absence of primary data on the health status of family members, it may be possible to draw on findings from related clinical trials or large‐scale datasets that have linked data on patient and family member health status. However, it is important to bear in mind that spillover effects are likely to be context‐specific and that direct measurement within the specific context of the research is likely to be the best solution.

In Table 7, we illustrate how the spillover estimates could be used to inform economic evaluation. 6Indeed a recent study has already used preliminary data on the sensitivity of family QALYs to patient health status to incorporate family health in their modelling of the cost‐effectiveness of vaccination against MenB (Christensen et al., 2014) The estimates of spillover health benefits (and hence total health benefits) are quite sensitive to assumptions made about the number of family members affected. However, the different methods for measuring health spillovers do result in similar conclusions. For example, the absolute spillover effect (0.041 QALYs per year) and relative spillover effect (16%) are broadly consistent with one another, when one considers that 16% of the annual QALY effect on survivors (0.19 QALYs) is 0.03 QALYs. It should be noted that this study focuses on measurement issues if these estimates are included in economic evaluation, they would need to be discounted in line with the assumption adopted in the economic evaluation.

**Table 7:** Using spillover estimates to project the total health benefits of preventing one case of long‐term after‐effects of meningitis

Input data	Spillover estimate (mean)	Estimating total health spillovers from intervention	Estimating total health benefits for economic evaluation	Notes
Impact of survivor morbidity on close family member health status [Absolute spillover].	0.041 QALYs per family member per year.	Assume annual QALY gain to close family members of 0.041. Multiply this by the number of close family members affected and years of benefit.	Aggregate survivor and family members' QALYs.	An assumption is needed about the number of family members affected.
**S**pillover coefficient extracted from regressing family member health status (*H_f_*) on survivor health status (*H_p_*) controlling for confounders [Relative spillover].	0.16 spillover from survivor health status to close familymember health status.	Apply multiplier of (*n* * 0.16) to any survivor QALY gains, where *n* is the number of close family members.	Apply multiplier of 1 + (*n* * 0.16) to survivor QALY gains.	An assumption is needed about the number of family members affected.
Two separate spillover coefficients extracted from regressing *H_f_* on *H_p_* within closest and second closest family member samples [Aggregate spillover].	0.18 spillover from survivor health status to closest family member, 0.11 to second closest.	Option 1: Apply multiplier of 0.29 (0.18 to 0.11) to survivor QALY gains.	Apply multiplier of at least 1.29 to patient QALY gains.	This assumes that only two close family members are affected. Estimate accounts for decline in spillover for second closest family member.
		Option 2: Extrapolate spillover effect to total family network using the empirical estimates.	Apply multiplier of 1.33 to 1.48 to survivor QALY gains.^a^	This assumes that spillovers extend to a wider family network, but estimate of these wider spillovers is made by extrapolating effect.
Spillover coefficients extracted from regressing of H_f_ on *H_p_* within sub‐groups of specific family members [Aggregate spillover].	0.14 spillover for survivors	Add coefficients for a representative close family network (e.g. two parents would be 0.28) and apply the resulting multiplier to survivor QALY gains.	Apply multiplier of 1 + (*X*) to survivor QALY gains, where *X* is the aggregate spillover effect for the representative family network.	Estimate of total health benefits takes into account composition of family networks.
	0.36 spillover for siblings			
	0.24 spillover for partners			

a Note: Using arithmetic progression the spillover effects in successive family members are 0.18, 0.11, and 0.04, resulting in an aggregate spillover of 0.33. Using geometric progression, the spillover effects in successive family members decline are (0.18, 0.11, 0.07, 0.04…), resulting in an aggregate spillover of 0.48.

This is the first study to compare methods for measuring health spillovers for use in economic evaluation. However, there are some limitations that are worth noting. First, we used family members' proxy reports of survivors' health status. Evidence suggests that parents may be reliable reporters of physical limitations in children, but their ability to report more subjective outcomes is more limited (Ungar, 2011). As noted earlier, proxy ratings of survivor health status were used for practical reasons and did not introduce bias in terms of the size of the spillover coefficients. Nevertheless, it would be interesting to see if the findings of this study would be replicated with patients that reported their own health status. Second, our categorisation of family members as closest and second closest used pragmatic criteria we developed based on the data collected in the study. Using other criteria, for example, bespoke instruments to measure family attachment, may well have resulted in a slightly different categorisation. Third, in the regression models, we attempted to control for the factors that could confound the relationship between the health status of family members and survivors. However, there may have been environmental factors, affecting both the health of survivors and family members, which we were not able to control for. It is important to note however that we purposefully avoided variables relating to informal care, as these may be on the causal pathway between the patient's health status and the family member's health status. Therefore, including such variables is likely to diminish the measured spillover effect.

There are a number of areas where further research is needed. We still know little about the scope of family impacts from healthcare interventions. The findings from this study suggest that spillover effects on two close family members are likely. However, the social network literature suggests that spillover effects may well spread more widely. Further use of ‘ecomaps’ (Rempel *et al.*, 2007), to depict patients' close family network, may be a useful way of identifying the scope of family impact prior to empirical work. The normative framework for considering health spillovers also requires consideration. Inclusion of health spillovers will most likely, although not always, inflate benefit estimates and therefore make interventions appear more cost‐effective (Goodrich *et al.*, 2012). Consequently, inclusion of spillover effects in cost‐effectiveness evaluations will potentially draw resources towards treatments for illness that have a higher impact on carers and family members and away from illnesses that affect only the patient. This may have important equity implications (Basu and Meltzer, 2005). Therefore, a sensible starting position might be to present spillover benefits alongside patient benefits, as well as in aggregated form, within an economic evaluation (Brouwer *et al.*, 1999, Goodrich *et al.*, 2012). This would enable policy‐makers to incorporate such information in decision‐making in a way they felt was appropriate.

This study demonstrates that sizeable health spillovers, which extend beyond the closest family member, are likely to occur in the context of preventing meningitis. From a methodological perspective, different approaches for quantifying health spillovers provided broadly consistent results. The choice of method will be influenced by the ease of collecting primary data from family members in intervention contexts. To align economic evaluation with improving *population*, as opposed to purely *patient* health, it is important that health spillovers are given attention in applied economic evaluation.

## Conflicts of Interest

The authors do not have any conflicts of interest to report in relation to this work.
